# X-linked vitamin D-resistant rickets: 12 years of follow-up

**DOI:** 10.11604/pamj.2018.30.9.14762

**Published:** 2018-05-04

**Authors:** Ahmed Anas Guerboub, Souad Moussaoui, Jad Issouani, Yassine Errahali, Ghizlaine Belmejdoub

**Affiliations:** 1 Endocrinology Diabetology and Metabolic Diseases Department of the Mohammed "V" Military Hospital, Rabat, Morocco

**Keywords:** X-linked hypophosphatemic rickets, PHEX, fractures and deformities

## Abstract

Rickets are abnormalities of mineralization that can lead to bone fractures and deformities. Vitamin-resistant rickets is defined as any rickets not prevented by regular, early and prolonged administration of vitamin D and not cured by a sufficient total dose. The aim of our work is to describe the clinical, paraclinical and therapeutic aspects of X-linked hypophosphatemic rickets (XLHR), which is the most common cause of hereditary rickets and on the other hand to highlight the interest not only of the early care but also the regular long-term monitoring of these children.

## Introduction

Rickets are mineralization abnormalities that can lead to fractures and deformities in the bone of a growing skeleton, as opposed to osteomalacia, an abnormality in the mineralization of an adult bone. Vitamin D deficiency, which was a major cause of rickets, is currently exceptional due to routine supplementation of newborns. The etiologies of rickets are diverse, including a variety resistant to this preventive therapy, called vitamin-resistant rickets, first described in 1937, previously considered as idiopathic [1]; and whose clinical and radiological presentation is identical to that of common deficiency rickets, but differs from the latter in severe hypophosphatemia and hyperphosphaturia. After the PHEX (phosphate regulating gene with homologies with endopeptidases on the X chromosome) protein was identified in 1995, the nomenclature became "X-linked hypophosphatemic rickets" (XLHR); which allowed to differentiate this form from other causes [2].

## Patient and observation

**Figure 1:**
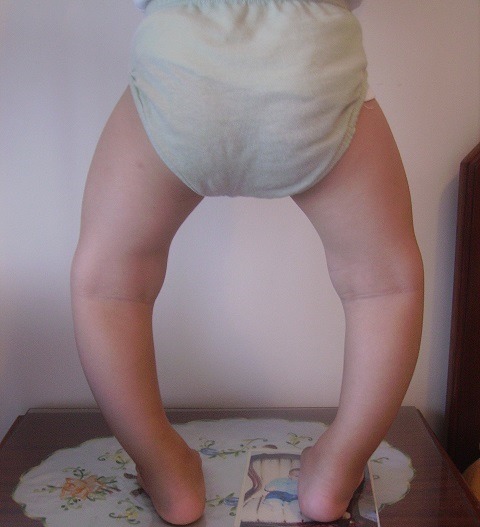
Photo at the age of 12 months

**Figure 2:**
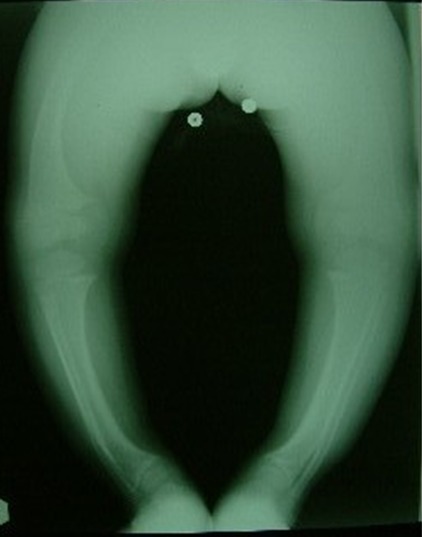
Imagery at the age of 12 months

**Figure 3:**
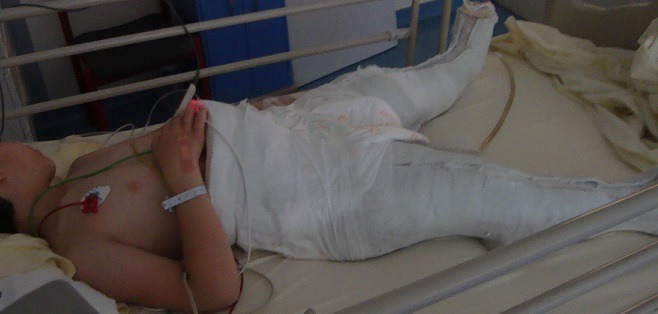
Photo in post-surgery

**Figure 4:**
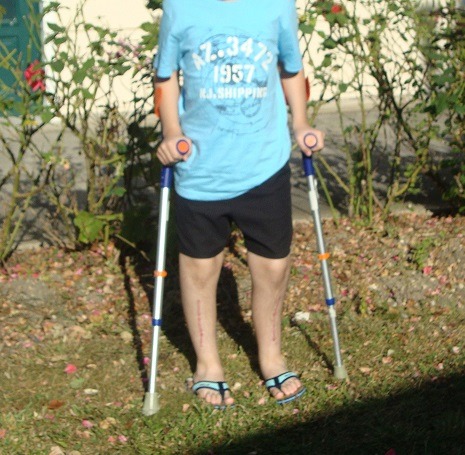
Photo at 3 months in post-surgery

Our observation illustrates the case of a child followed for 12 years for XLHR in the endocrinology and diabetology department of the Mohammed V military hospital in Rabat. This is the 16 year old child, single son, schooled. For antecedents: pregnancy was followed and completed with a birth weight of 4kg, a birth height of 51cm and a cranial perimeter at birth of 35cm with a non-specific neonatal period. There is therefore a consanguinity between maternal grandparents and maternal great-grandparents, a notion of small family size, namely a mother of 1.52m in height and a maternal uncle of 1.60m, whereas the father measures 1.80m and the target size of this child was 1.72m. The history of the disease goes back to the age of 2 years, this child is received in consultation for deformations of the lower limbs. According to the child's health booklet, stature growth was steady (+ 2DS) until the age of 12 months when a break occurred and passage on -2 SD and since steady growth on -2 DS. For weight, there is a gradual decline of +1 DS in the mean. On clinical examination we find a bilateral Genu Varum, radiology confirms the diagnosis (Genu Varum bilateral and symmetrical femoral and tibial) and highlights metaphyseal bulges, costal rosary and signs of osteoporosis despite adequate prophylaxis by the vitamin D (Figure 1, Figure 2). The biology eliminated tubulopathy and rickets with hypercalciuria, hypophosphatemic pituitary rickets confirmed with hypophosphoremia at 24 mg/l, hypocalciuria at 0.32 mg/kg/24h and alkaline phosphatase at 873 mg/ml and a decrease of 1 25 dihydroxy vitamin D at 17ng/l. In addition, a renal ultrasound eliminated nephrocalcinosis. The genetic study, carried out in 2010, objectified the presence of an insertion in exon 20 of 5bp, causing a shift of the reading frame, and premature stop codon: X-linked hypophosphatemic rickets with mutation of the PHEX gene. This child has been treated for 5 years with: Phosphoneuros^®^ (drops) and alpha^®^ (drops and capsules) with a dose adjustment based on calciuria and alkaline phosphatase levels. The evolution was marked by the persistence of Genu Varum, the appearance of osteopenia and a slowdown in regular growth at -2 SD, hence the introduction of growth hormone, as well as recurrent dental abscesses (more than 15) requiring many dental avulsions. Due to worsening bone deformities and stunting, surgical treatment was indicated. Thus, a medio-diaphyseal femoral and tibial osteotomy of valgization (femur) and bilateral valgation-derotation (tibia) was performed in June 2012 with immobilization platerée pelvi bi pedieux for 45 days. Follow-up is marked by paresis of the posterior tibial nerve with progressively regressive evolution and verticalization is performed from J30 with plaster and J45 without plaster, with additional medical treatment (Figure 3, Figure 4). The disease has been biologically and radiologically stable for growth, the current size is -1 SD and the final prognosis for this child is less than 1.65m. Currently, the patient weighs 51kg with a height of 1.66m and under the following treatment: Phosphoneuros^®^ (85 drops 3 times a day), growth hormone (Norditropine Simplexx^®^ 3.5 mg/day 6 days a week), alpha^®^ (4 μg/day) and Uvedose ^®^ 100,000 IU of light bulb every 3 months.

## Discussion

X-linked hypophosphatemic rickets is the most common cause of hereditary rickets associated with renal phosphate loss. The phenotype is characterized by selective renal phosphate loss due to abnormal phosphate tubular reabsorption, abnormal phosphate and calcium intestinal absorption and skeletal mineralization defect. These abnormalities are not corrected by physiological doses of vitamin D. The disease is more common in women, but more severe in men. In children, the most common group of renal phosphorus re-uptake disorders is XLHR, which has an estimated incidence of 1 per 20,000 [2, 3]. XLHR is a hereditary disease, inherited predominantly by sex, but other modes of transmission, recessive, have been reported. The HPDR locus (responsible for XLHR) is located on the distal part of the short arm of chromosome "X" in region Xp 22. 1-p 22.2 between markers DXS-41 and DXS-43 [4]. The affection can be transmitted [5]: from a mother to her sons or daughters: one in two children can be reached; from a father to his daughters only: all the girls are then inevitably affected. As a result, rickets are twice as common in girls as in boys, but they are also less severe in their clinical and biological symptoms. In most cases where the child is sporadically affected by primary hypophosphatemic rickets, this is likely due to a spontaneous mutation of the affected X chromosome gene. The following generations then transmit the disorder under the X-linked dominant mode [6]. The findings on the PHEX gene and its mutations in XLHR patients were an interesting development. In 1995, the gene responsible for XLHR was cloned. PHEX is a type II membrane protein of the family of zinc metallo-endopeptidases whose role is to cleave and therefore inactivate the fibroblast growth factor phosphatonin (FGF) 23. The result of this mutation is therefore the accumulation of FGF23, increased phosphaturia and chronic hypophosphatemia. To date, more than 200 different mutations of the PHEX gene have been identified [7, 8]. This form of rickets can be divided into 4 clinical entities of variable intensity and expression of the disease [9]: asymptomatic hypophosphatemia: girls are particularly prone to this clinical form. In this case, evidence of the metabolic defect requires biological evaluation and possibly evaluation of the tubular reabsorption rate in phosphorus; hypophosphatemia in adults: where post-rachitic deformities remain non-progressive; adult hypophosphatemia: where deformities and osteomalacia remain active; hypophosphatemia of the child: this entity is clinically the most severe as our observation illustrates.

Conventional treatment will be essentially medical. It is based on the combination of oral supplementation of inorganic phosphorus (1 to 3.6 g per day in several doses) and calcitriol (0.5 to 2 μg per day). The dose will be adjusted according to the severity of rickets, the response to treatment, the complications encountered. Medical treatment has beneficial effects on stature growth, phosphate homeostasis and rickets. However, without a satisfactory explanation, some patients do not grow normally as for our patient. Many studies suggest that the response to conventional treatment in terms of growth does not depend on either the biochemical response to treatment or the deformities of the inner limbs and that the main criterion for predicting the quality of stature growth would be the size at the start of treatment [10]. Also, we note that the average height of the parents influences the final size of the child, but only for girls. Heterozygous girls appear to respond better to conventional treatment than hemizygous boys, suggesting that the genesis of small size in XLHR patients is therefore likely to be multifactorial [11]. Although the low growth rate observed in XLHR does not appear to be related to growth hormone abnormality, long-term GHr (Human Growth Hormone) hormone therapy associated with conventional therapy may improve growth prognosis and Radial bone mineral density [12]. Corrective surgery can be performed according to the importance of bone deformities. Despite the improvements brought by medical treatment, surgical treatment remains an essential remedy for correcting severe deformities of the lower limbs. It is generally accepted that osteotomy is justified in childhood only if the arcature is so severe that it can only worsen during growth, despite the medical treatment as for our case. Therefore, the surgical indication in the XLHR arises case by case, depending on the importance of the deformations of the lower limbs. After surgery, dosage adjustment of calcitriol and phosphates is required due to hypercalcemia induced by immobilization. The therapeutic use of anti-FGF 23 antibodies is currently being tested after having been shown to be effective in the animal model of HYP mice [13]. Given the frequency of tooth abscesses related to tooth structure anomalies, they are an integral part of XLHR management. The standard treatment of abscesses by pulpectomy and occlusion of the dental canal is not always satisfactory. Prosthetic crown tooth protection is an effective prophylactic measure. We see that all dental care must be done by a dentist who is familiar with the specificities of XLHR [14].

## Conclusion

Several studies will have to be undertaken to determine the physiological role of PHEX in a disease such as XLHR, as this may help us better understand its role in mineralization. The continuation of these studies would imply the use of new specific therapies to optimize the multidisciplinary management of the XLHR which must be early with a regular follow-up in the long course to improve the prognosis.

## Competing interests

The authors declare no competing interests.
